# Electric Field Detection in Sawfish and Shovelnose Rays

**DOI:** 10.1371/journal.pone.0041605

**Published:** 2012-07-25

**Authors:** Barbara E. Wueringer, Lyle Squire Jnr, Stephen M. Kajiura, Ian R. Tibbetts, Nathan S. Hart, Shaun P. Collin

**Affiliations:** 1 The University of Queensland, School of Biomedical Sciences, Brisbane, Queensland, Australia; 2 The University of Western Australia, School of Animal Biology and the UWA Oceans Institute, Crawley WA, Australia; 3 Cairns Marine, Stratford, Queensland, Australia; 4 Biological Sciences, Florida Atlantic University, Boca Raton, Florida, United States of America; 5 The University of Queensland, School of Biological Sciences, Brisbane, Queensland, Australia; 6 The University of Queensland, Queensland Brain Institute, Brisbane, Queensland, Australia; Ecole Normale Supérieure de Lyon, France

## Abstract

In the aquatic environment, living organisms emit weak dipole electric fields, which spread in the surrounding water. Elasmobranchs detect these dipole electric fields with their highly sensitive electroreceptors, the ampullae of Lorenzini. Freshwater sawfish, *Pristis microdon,* and two species of shovelnose rays, *Glaucostegus typus* and *Aptychotrema rostrata* were tested for their reactions towards weak artificial electric dipole fields. The comparison of sawfishes and shovelnose rays sheds light on the evolution and function of the elongated rostrum (‘saw’) of sawfish, as both groups evolved from a shovelnose ray-like ancestor. Electric stimuli were presented both on the substrate (to mimic benthic prey) and suspended in the water column (to mimic free-swimming prey). Analysis of around 480 behavioural sequences shows that all three species are highly sensitive towards weak electric dipole fields, and initiate behavioural responses at median field strengths between 5.15 and 79.6 nVcm^−1^. The response behaviours used by sawfish and shovelnose rays depended on the location of the dipoles. The elongation of the sawfish’s rostrum clearly expanded their electroreceptive search area into the water column and enables them to target free-swimming prey.

## Introduction

Elasmobranchs use electroreception to navigate in the earth’s magnetic field and to detect inanimate objects and living organisms such as predators, prey and conspecifics [1]. Scalloped hammerhead sharks *Sphyrna lewini* are thought to follow the geomagnetic field lines during their diurnal migrations to and from seamounts in the Pacific Ocean [2]. Johnson et al. [3] conditioned juvenile nurse sharks *Ginglymostoma cirratum* to successfully detect and retrieve metallic spheres in a tank in the presence of a background electric field. Round stingrays, *Urolophus halleri* use the electric field produced by buried conspecifics to orient themselves in order to optimize social interactions in the mating season [4]. Moreover, embryos of clearnose skate *Raja eglanteria* display a freeze response while still encased in their egg purse, and cease all ventilatory movement during the approach of an external electric field resembling a predator [5].

In the context of prey capture, electroreception provides elasmobranchs with the ability to precisely locate prey and hunt in both the dark and/or in turbid waters, opening up a rich ecological predatory niche [6]. Naturally occurring localized dipole sources in the aquatic environment only originate from living animals and therefore their presence equates to the presence of another organism [7]. Various species of sharks and rays have been shown to readily attack weak electric fields both in captivity and under natural conditions [1], [8]–[12]. The electro-location task can be divided into three components, i.e. detection, characterization and localization [6]. These processes can be described separately although they are tightly coupled and synchronous in the nervous system [6].

Electroreceptive cues differ from stimuli passively perceived with other sensory organs, as they do not provide the receptor with a temporal component or propagation velocity vectors. The frequencies of biologically important electrosensory cues range from almost DC to a few 100 Hz, resulting in a wavelength of several kilometres [13]. As a result, electric fields propagate with nearly infinite speed, and are present throughout their full extent almost instantaneously [13], [14]. The biologically important characteristics encoded in electric stimuli are the local intensity, orientation and the polarity of a field [14]. Electric flux lines describe a curved path along the direction of the current and do not point straight to their source. Behavioural experiments indicate that the stimulus frequency ranging from DC up to 8 Hz has little, if any significance for behaviour, as electroreceptive predators attack artificial dipoles provided as long as their frequencies are within the detectable range [14], [15].

Physiologically, the ampullae of Lorenzini, which are the electroreceptors of elasmobranchs, are not true DC receptors, and this characteristic is important for their normal mode of operation within the animal’s own DC background field [1], [14], [15]. In order to sense the DC field produced by prey, elasmobranchs must move with respect to their prey [1], [14], [15].

Here, we compare the electroreceptive abilities and behaviours of a sawfish (*Pristis microdon*) and two shovelnose rays (*Aptychotrema rostrata* and *Glaucostegus typus*), which all evolved from a rhinobatid-like ancestor [16]–[19]. The morphology of the ampullary systems of all three species is known [20], [21]. *P. microdon* possesses one of the highest numbers of electroreceptors of any elasmobranch (and twice as many pores as *A. rostrata* and *G. typus* [20]) and may thus be considered an electroreception specialist [21]. They possess more ampullary pores ventrally than dorsally, but dorsally pores are concentrated along the rostral cartilage [21]. In both species of shovelnose rays, ampullary pores on the ventral side of the head are about five times more numerous than on the dorsal side, with the highest concentrations on the ventral rostrum [20].

Even though commonly referred to as freshwater sawfish, juvenile *P. microdon* occupy oligohaline to mesohaline low visibility environments, while adults may move into saltwater [18], [22]. Consequently electroreception may be especially important for the detection and manipulation of prey by juvenile sawfish. Their diet is dominated by benthopelagic teleosts and prawns of the genus *Macrobrachium* spp. [22]. We thus hypothesise that freshwater sawfish will orient towards artificial electric fields and that they will be able to detect and respond to dipoles presented in the water column, whereas rhinobatid shovelnose rays will not. Rhinobatid shovelnose rays tend to inhabit clearer water and forage on benthic invertebrates. The diet of *G. typus* is dominated by brachyuran crabs and penaeid shrimp, which make up more than 50% of the relative importance of food items in animals below and above 150 cm TL [23]. The diet of *A. rostrata* is dominated by penaid prawns and carid shrimps, which make up more than 50% of the index of relative importance [24].

## Methods

### Study Species

#### Freshwater sawfish *Pristis microdon*

Nineteen juvenile freshwater sawfish *Pristis microdon* (12 males and 7 females, total length between 96.0 and 208.0 cm) were captured from their natural habitat in far North Queensland (Norman River, S 17°38′, E 141°0′, Wenlock River, S 12°16′, E 141°58′) and transported to the holding facility of Cairns Marine (Cairns, Queensland, Australia) according to the company’s protocols and approval by the UQ Animal Ethics Committee. In general, sawfish began to feed after three days in captivity and were given at least another three days of acclimation before experiments commenced.

The holding tanks, which also served as experimental tanks, were made of fibreglass (diameter of 4 m, water depth of 80 cm). The tanks rested on timber, which insulated them from the ground. The water pump was earthed. Three to five sawfish were kept together in each tank depending on their size. To minimize stress in animals and also to minimize handling of potentially dangerous animals, sawfish were not separated for behavioural trials.

#### Rhinobatid shovelnose rays

Shovelnose rays belonged to two species, namely the giant shovelnose ray *Glaucostegus typus* (20 juveniles, total length between 38.0 and 180.0 cm) and the eastern shovelnose ray *Aptychotrema rostrata* (7 juveniles and adults, total length between 44.0 and 76.5 cm). They were caught in marine environments off Heron Island, Shark Bay (S 23°31′, E 152°1′), off Adams Beach, North Stradbroke Island (S 27°29′, E 153°24′) and off Double Island (S 16°43′, E 145°41′), Queensland, Australia. All specimens were fed to satiation after capture and then starved for 3 to 5 days before trials commenced to render them in the same nutritional state. Animals were tested in the holding tanks of the Heron Island Research Station (dimensions 4×4 m, water depth 50 cm), the Moreton Bay Research Station (diameter 4 m, water depth 120 cm) and Cairns Marine (diameter 4 m, water depth 80 cm). All specimens caught on Heron and North Stradbroke islands were released after trials ended (up to 28 days after capture).

### Stimulus Delivery

**Figure 1:**
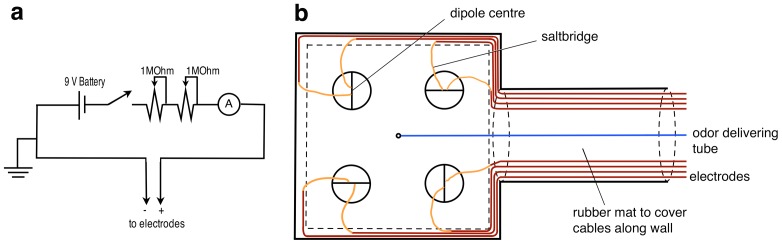
The experimental set up. (**a**) The electric circuit that delivered a low electric current into the experimental tank via electrodes. The strength of the current was varied with the two variable resistors (**b**) Schematic drawing of the transparent rubber mat on which dipoles were mounted that delivered electric fields on the bottom of the tank. Double layers of rubber are indicated with dashed lines. Cables were protected by these layers to prevent entanglement of the animals. The two plastic tubes of one dipole were oriented approx. perpendicular to the centre of the dipole to avoid the build up of electric fields along the tubes.

The current-delivering device was modified after Kajiura and Holland [10]. An electric circuit (figure 1a) delivered a constant electric current. The strength of the current was varied with two variable resistors. A multimeter (Fluke Electronics, 116 HVAC Multimeter) was connected in series to measure the generated electric current. Each electrode was made of a silver pin soldered to a shielded coaxial cable (RG58C/U, Dick Smith Electronics) that was plugged into the stimulus generator. The cable/silver interface was shielded with marine silicone and shrink tubes so that only the 5 mm long tip of the silver pin was in contact with the water. Each silver pin extended into a transparent plastic tube (length 50 cm, inner diameter 3 mm) filled with seawater, which acted as a salt bridge. Both the shield of the cable and the electric circuit were earthed.

Ten specimens of *Glaucostegus typus* were tested with a slightly different experimental set up that used 5 mm long electrode tips made of silver coated with chlorox solution (Ag/AgCl) to minimize polarisation currents, but not salt-bridges. Electrodes were recoated between trials, and the two electrodes of a dipole were spaced 1 cm apart.

#### Electric fields presented on the substrate

The experimental set up was modified after Kajiura and Holland [10]. Four dipoles made of eight electrodes were mounted on the underside of a rubber mat, with only their ends protruding through the rubber (Clark Rubber, 138 cm ×138 cm, 1 mm thick, see figure 1b). The distance between the two electrodes of a dipole was 2 cm and the distance between dipoles was 50 cm. The distance of 2 cm between electrodes was chosen as it resembles the distance between the gills of potential teleost prey [25]. A black circle (radius 10 cm) was drawn around each dipole and the dipole axis was marked with a black line, to visualise the dipole centre on video footage. A plastic tube (inner diameter 3 mm) was mounted in the centre of the rubber mat, equidistant to all dipoles. It delivered an olfactory cue made of blended and filtered mullet chum via a syringe, which induced food searching behaviour and also attracted the rays to the rubber mat. The rubber mat also extended onto the tank wall, covering the coaxial cables and the rubber tube to prevent entanglement of the animals.

#### Presentation of electric fields in the water column

The dipole centres were suspended in the water column approximately 20 to 30 cm above the substrate. The same stimulus device was used but dipoles were hung from a wooden pole that was laid horizontally across the tank above the water line. To ensure even spacing of the two plastic tubes of a salt-bridge, a plastic tube with an outer diameter of 2 cm was placed between them. The three tubes were held in place, protected and surrounded by a fourth plastic tube, on which a black line was drawn 10 cm from the dipole. The two dipoles (one active and one control) were separated by 130 cm and a plastic tube (inner diameter 3 mm, length 5 m) extended to the same depth into the water half way between them. This tube delivered chum via a syringe.

### Experimental Procedure

The experimental set up was lowered into the tank at random times to get animals accustomed to its presence and to avoid pseudo-conditioning and associations of the set up with either food or electric fields. Animals were tested twice a day before feeding, and each experimental session lasted 40 mins. During behavioural trials only one dipole was active, while the three/one other dipoles served as controls. Trials with dipoles on the bottom were filmed with one remotely controlled video camera (Sony, DCR HC96E or DCR HC5) in a water resistant case (Sony, SPKHCB) mounted above the tank. Trials in which dipoles were suspended in the water column were filmed with two cameras, one mounted above the tank and the other one behind a camouflaged window in the tank wall. A trial lasted for 5 mins and the active dipole was switched randomly between trials.

The electric current used was also randomized and noted for each trial. Sawfish were tested with currents of 18.9–50.0 µA and 21.6–80.3 µA for electric fields presented on the bottom and in the water column, respectively. Shovelnose rays were tested with currents of 11.7–28.4 µA for electric fields on the bottom. Currents that tested the response of *G. typus* towards electric fields in the water measured 24.3–90.7 µA. Shovelnose rays were tested in saltwater (36.4–38.6 ppt), while sawfish were tested in brackish and saltwater (15.5–35.2 ppt). After each experimental session all animals were fed to satiation. Before and after each experimental session, water temperature, salinity and conductivity were measured with a TPS WP-84 conductivity meter.

Shovelnose rays were only tested in natural light conditions, while sawfish were also tested in infared light, to test the possibility that visual stimuli were responsible for any orientation responses. As inactive dipoles also served as visual controls, we hypothesized that the infrared light condition would not influence the results. For infrared trials, the tank was covered with two layers of black plastic sheeting (thickness of one layer 200µm). Four infrared lights (Jaycar Electronics QC3652, each with 56 units of IR LEDs emitting light of 850 nm wavelength which is near infrared light) were mounted 5 cm above the water surface. Trials were filmed with an infrared sensitive security camera (Jaycar Electronics digital CCD Camera) mounted above the tank. Illuminance was not measured directly but as visible on video clips, the camera switches from normal to infrared mode automatically at light levels between 0.24 and 0 LUX. These light levels correspond to natural light levels measured in air during deep twilight (1 LUX) and full moon (0.1 LUX).

### Analysis of Results

Videos of experiments were digitized using iMovie HD 6.0.3. When viewing these clips, behavioural units that the animals displayed during each response towards a dipole were noted and characterized. Behavioural units were defined after Barlow [26]: a modal action pattern is a natural, stereotypic unit of behaviour that contains a variable, taxic component. Therefore, the modal action pattern describes a spatio-temporal pattern of movement that cannot be further subdivided [26]. The descriptive ethogram that was established for each species is described in detail elsewhere [27].

To estimate the detection threshold, the strength of the electric field at the point of initiation of a behavioural orientation response ( = PIR) was estimated after the method of Kajiura and Holland [10]. PIR is defined as the point where an animal initiates a turn of more than 20° towards the dipole. The respective frame was saved from each video clip. In ImageJ 1.39u (http://rsb.info.nih.gov/ij/) the following measurements were taken: the minimal distance *r* [cm] from the centre of the dipole to the side of the head, taking into account the distribution of the ampullae of Lorenzini [20], [21], and the angle α [°] between that point and the dipole axis. The electric field strength E [nVcm^−1^] at PIR was calculated using Maxwell’s [28] electric field equation, Ε = (ρ.*I.d*. cosα)/(π.*r* ^3^), in which ρ is the resistivity of the water [Ω cm], *d* is the distance between the two electrodes of a dipole [cm] and *I* is the electric current [µA]. The equation was altered for the calculation of the field strength at PIRs towards dipoles located in the water column, where the field resembles a sphere. The distribution of data points for each treatment was tested with a Kolmogorof-Smirnov test for normality with Lilefors correction or a Shapiro-Wilk test for normality. As field strengths at PIR for all treatments were skewed to the right, non-parametric methods were used for analyses.

Orientation responses during which multiple animals were visible in the frame were excluded from analysis, to avoid misjudgement of unrelated behaviours as orientation responses. Only the first orientation response was analysed if an animal repeatedly returned to a dipole after an initial bite without leaving the frame. Moreover, only turns that resulted in further reactions towards the dipole were analysed, to exclude randomly initiated turns from analyses.

How often animals passed within 10 cm of a dipole on the bottom (categorized as active, left of active, right of active and opposite of active dipole) or touched a dipole in the water column (categorized as active and inactive) without displaying any reaction was noted together with the trial duration and current. Frequencies were analysed with Chi^2^ and binomial tests to determine if animals searched all electrodes equally or if they passed over the active dipole more often than predicted by chance alone. All statistical procedures were completed using Microsoft Excel 12.1.3 and PAWS 18.0 and follow Sokal and Rohlf [29].

## Results

Reactive sequences are distinguished based on the location of the dipoles, which can be on the bottom (*P. microdon* n = 146, *A. rostrata* n = 60, *G. typus* n = 26), or in the water column (*P. microdon* n = 57, *G. typus* n = 60). An additional 134 reactions were recorded for *G. typus* reacting towards dipoles made of Ag/AgCl electrodes presented on the bottom. Inactive dipoles presented on the substrate were bitten once by *P. microdon* and once by *G. typus*. Three times, sawfish bit the end of the bottom chum tube after passing it when the mullet chum was released into the water. *P. microdon* did not react to inactive dipoles suspended in the water column. The reactions of *G. typus* towards dipoles in the water column are discussed separately.

### Behavioural Analysis

**Figure 2:**
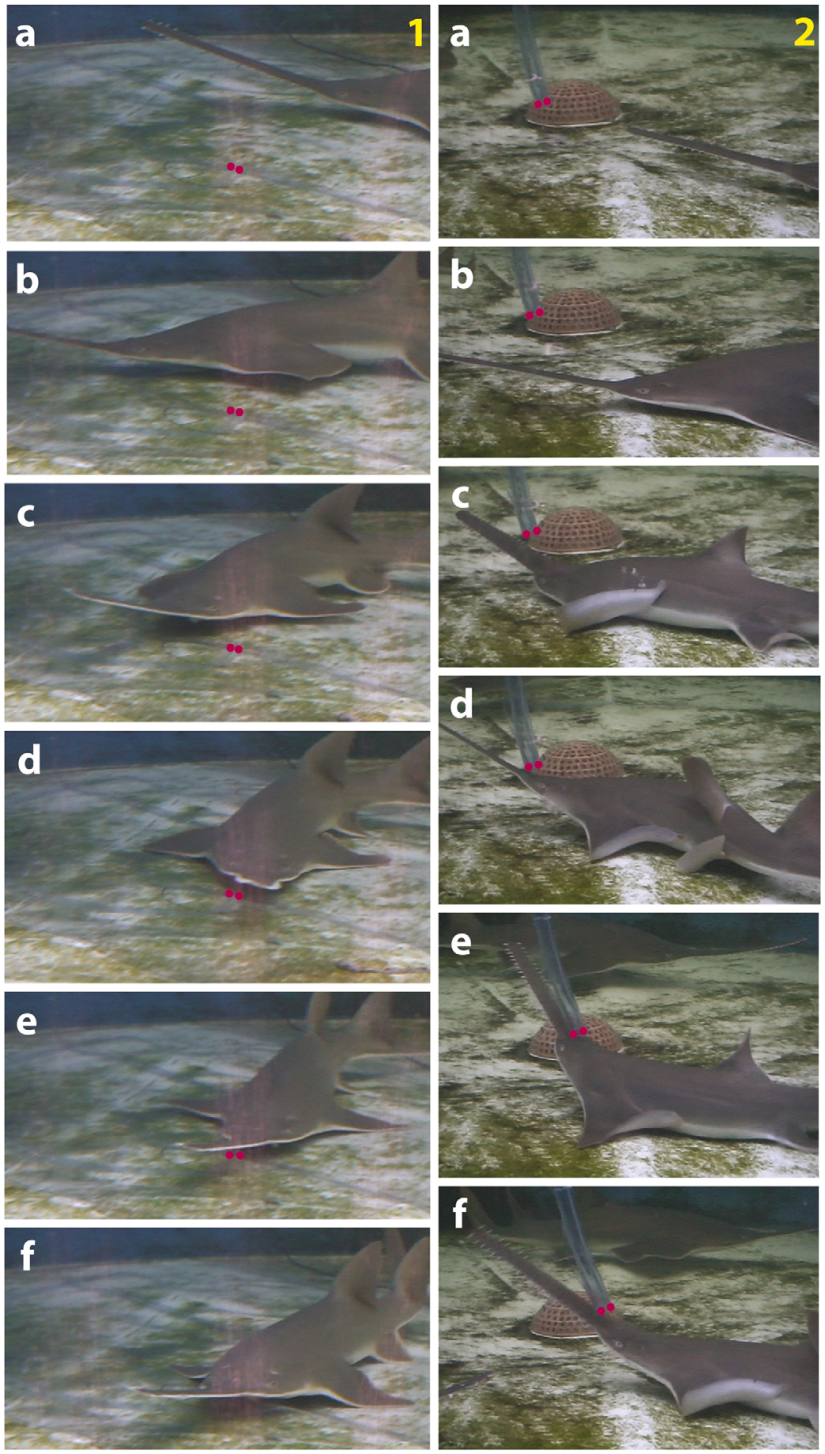
Reaction sequence of juvenile sawfish towards electric dipoles. Dipoles (indicated with two red dots) are presented (1) on the substrate and (2) in the water column. The sawfish swims past the dipole and turns towards it (1a–b), wiggles the rostrum over the dipole centre (1c–d), approaches further (1e) and bites the dipole centre (1f). When reacting to the dipole suspended in the water, the sawfish almost passes the dipole (2a–b), turns towards it (2c) and produces ‘saw in water, (2d–f).

The modal action patterns that sawfish and shovelnose rays displayed are described in detail elsewhere [27], and are only mentioned briefly here with their occurrence probabilities (p_o_). The behaviours that sawfish displayed when attacking the active dipole depended on the location of the dipole (figure 2). Sawfish bit dipoles on the bottom (p_o_ = 0.90, figure 2 section 1) but produced fast lateral swipes aimed at dipoles located in the water column (p_o_ = 0.81, figure 2 section 2). The only modal action pattern to occur in both treatments is *wiggle* (on bottom p_o_ = 0.10, in water column p_o_ = 0.19). During *wiggle* a sawfish produces a slight lateral movement of the head.

When reacting to an active dipole on the bottom, shovelnose rays always bit its centre (p_o_ = 1.00). *Glaucostegus typus* displayed two modal action patterns towards dipoles suspended in the water column that were neither displayed towards bottom dipoles nor by sawfish, namely *repeated bumps* (p_o_ = 0.29, the dipole is repeatedly bumped into with the rostrum) and *spiral* (a shovelnose ray swims in a circle while maintaining contact between the side of the head and the dipole). *Spiral* was displayed both towards the active (p_o_ = 0.59) and inactive dipole (p_o_ = 0.12).

### Detection Threshold of the Electric Field

The median electric field strength (± std. dev.) at PIR of *P. microdon* for electric fields located on the bottom was 13.4±700.1 nVcm^−1^ (n = 118). The median electric field strength at PIR for dipoles suspended in the water equalled 10.0±166.2 nVcm^−1^ (n = 18). The median electric field strengths at PIR did not differ significantly between the treatments (dipoles on bottom vs. dipoles in water column; Mann-Whitney U-test, n = 118, z = −0.76 p = 0.45) and so the two subsamples were pooled for further analysis. The median field strength at PIR of the pooled data was 13.0±640.0 nVcm^−1^. There was also no significant difference between the electric field strength at PIR in daylight or infrared light trials (Mann-Whitney-U test, n = 118, z = −1.74, p = 0.082).

Juvenile freshwater sawfish were tested for their reactions towards weak electric fields in salinities between 15.5 and 35.2 ppt, with a mean salinity of 28.3±4.8 ppt. To examine a possible correlation between the field strength at PIR and salinity, data were sorted by the following groups: 15–20 ppt, 20–25 ppt, 25–30 ppt, 30–35 ppt. Field strength medians were compared between groups via a Kruskal-Wallis test, which was corrected for tied ranks. The results indicate that there is no significant difference between the medians (X^2^ (3, N = 118) = 3.079, p = 0.38) of different salinity treatments and therefore data were pooled.

The median electric field strength at PIR for electric fields located on the bottom is 79.62±126.51 nVcm^−1^ for *A. rostrata* and 25.33±424.78 nVcm^−1^ for *G. typus*, respectively. The median electric field strength at the PIR for ten specimens of *G. typus* reacting towards electric fields on the bottom produced by Ag/AgCl electrodes is 5.15±202.9 nVcm^−1^, which is significantly lower than the median field strength at PIR for reactions towards dipoles made of salt bridges (Mann-Whitney U-test, p<0.01). No electric field strength at PIR could be calculated for responses by *G. typus* towards electric fields suspended in the water, since the reactions were quite erratic and a point of initiation of the response could not be determined.

The median electric field strength at the PIR was compared between test species for electric fields located on the bottom produced with salt bridge electrodes. The median field strength (median test, median = 17 nVcm^−1^, df = 2, n = 141, p = 0.051) does not differ across species.

When sawfish grow, the length of ampullary canals increases [21]. As longer canals create a larger potential difference between the inside and outside of the animal, longer ampullary canals are predicted to increase the animal’s sensitivity to any electric field [30]. To test this prediction, animals in analysis videos were assigned to one of three different size classes (small, TL 96–132 cm, medium TL 142–159 cm, large TL 178–208 cm). However, neither the distribution of the detection threshold electrical field strengths nor the median detection threshold electric field strengths differed significantly among the groups (df = 2, n = 112, Kruskal-Wallis test p = 0.080, median test p = 0.401).

### Correlation of the Distance between the PIR and the Angle to Dipole Axis

**Figure 3:**
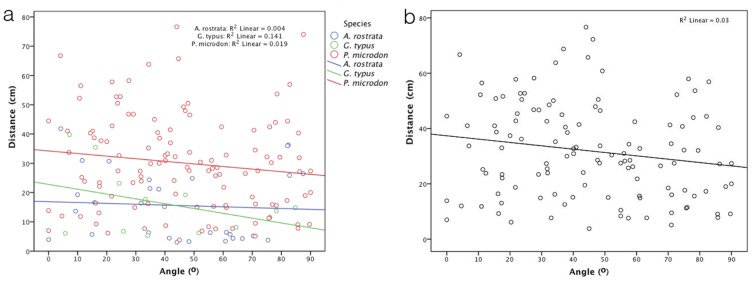
Scatterplot of the orientation distance [cm] plotted against the orientation angle [°] at PIR towards dipole electric fields. (a) Values measured for reactions by sawfish *Pristis microdon* and shovelnose rays *Aptychotrema rostrata* and *Glaucostegus typus* towards electric dipoles presented on the bottom, and (b) for *P. microdon* towards dipoles located on the bottom and in the water column. The orientation distance decreases slightly at higher angles, but this relationship is not significant (see text for statistical analysis).

The electric field around a dipole is not uniform, as the electric flux originates in the positive electrode and ends in the negative one. Equipotential surfaces are perpendicular to the lines of electric flux. As a result the electric field around a dipole at a given distance from the dipole is strongest at a small angle to the dipole axis and theoretically reaches zero perpendicular to the dipole axis. A linear regression was performed to test if the approach angle could predict the distance at PIR. However, the distance between the dipole and the PIR was less than 3% predictable by the angle of approach when all treatments (bottom and water column, four salinity groups) were pooled (R^2^ = 0.03). Therefore the approach angle does not significantly influence the distance at which an orientation turn is initiated (ANOVA, F(1,116) = 2.2, p = 0.14). Data separated by the treatments did not show correlations either (figure 3a and 3b).

No strong correlation was found between the distance at PIR and the angle between the PIR and the dipole axis, as indicated by the Kendall τ correlation coefficient (*A. rostrata* τ = −0.06, not significant at p = 0.63; *G. typus* τ = −0.21 not significant at p = 0.33). However, a small but slight trend is visible for all species, in that the distance of PIR is larger at smaller angles (see Figure 3).

### Passes Under Dipoles

There was no significant difference in how often sawfish passed over active and inactive dipoles on the bottom (Chi^2^ Test, p = 0.20, df = 3, n = 118), or how often they touched the touched active and inactive dipoles in the water column (Binomial test, n = 45, p = 0.37). *Glaucostegus typus* did not pass over any dipole on the bottom more frequently than predicted by chance (binomial test, p = 0.62, df = 36), and neither did *A. rostrata* (binomial test, p = 0.1, df = 75). Accordingly, there was no difference between the frequencies of *G. typus* passing under or touching the active or the inactive dipole suspended in the water column (pass under dipoles: binomial test, p = 0.24, df = 58; touch dipoles: binomial test, p = 0.24, df = 18).

## Discussion

The specific uses of the different sensory modalities of elasmobranchs are better understood when classifying them according to their detection ranges, which are determined by the sensory capabilities, the stimulus strength and the physical characteristics of the environment [31]. Both olfactory and acoustic stimuli are detectable over long ranges and can attract sharks from large distances. Gustation and touch are close range senses that need physical contact for stimulus detection [31]. Electroreception, vision and mechanoreception function at medium range, and are thus useful in prey capture. Within the medium range senses, the effective detection distance of vision in the underwater environment is the furthest, as it can be up to 100 m depending on the habitat visibility. Mechanoreceptive stimuli are detected by fish within a distance of about one body length [32]. The detection ranges of electroreceptive stimuli depend on the stimulus strength. Sharks and rays respond to prey simulating electric fields from distances of around 40 cm, which corresponds to stimulus strengths of the order of 1 nVcm^−1^ to 1 µVcm^−1^ [10], [28].

In this study, an olfactory stimulus was introduced into the tank to elicit food-searching behaviour. As the chum delivering tube was located some distance from the centre of the dipoles and was rarely attacked, we do not consider that olfaction was used to locate the source of the electric field. This is emphasised by the fact that sharks (*Carcharhinus menisorrah  =  falciformis*) need an additional stimulus to induce feeding, after the location of the source of an olfactory cue has been confirmed [33].

A turn of more than 20° was assigned to be a ‘single turn’, following the criteria of Kajiura and Holland [10] for comparative purposes. The angle of 20° is clearly discernible to the observer and is larger than the turns that sawfish and shovelnose rays make during normal swimming movements. The location from where an animal approaches the active dipole is guided by chance, but also depends on the prevailing water currents in the tank that distribute the olfactory cue. Moreover, if multiple animals are present in the tank, they tend to synchronize their swimming direction.

Shovelnose rays and sawfish both responded equally to bottom dipoles with a bite. The bite indicates the importance of electroreception in guiding an elasmobranch to its prey. To date, all elasmobranchs tested for their response towards electric dipoles displayed either a biting response or produced suction feeding movements [1], [8]–[11], [15], [28], [34]–[36], but freshwater sawfish also produce lateral swipes of their rostrum towards dipoles suspended in the water column [27]. Benthic skates are highly electroreceptive [37]–[40], but *Raja erinacea* failed to react to the vertical component of an electric field: when presented with a dipole above the pectoral fins, the animals instead attacked a location on the substrate under the pectorals fin directly below the centre of the suspended dipole, apparently unable to interpret a signal with a vertical component [7]. Thus sawfish, which are generally considered to be sluggish benthic dwellers [41], may actually be agile, demersal predators.

The occurrence of lateral swipes of the rostrum aimed at the active dipole, and of *wiggle* in sawfish and not in shovelnose rays indicates that these behaviours may have evolved in relation to the evolution of the elongated rostrum [27]. The displays of *spiral* and *repeated bumps* by shovelnose rays may indicate reactions to electric dipoles in the water column, but the lack of skills to manipulate them, as these skills evolved in sawfish together with the elongation of the rostrum [27]. Thus, *repeated bumps* displayed by *G. typus* towards dipoles in the water column may be an uncoordinated predecessor of *wiggle*, a behaviour from which *wiggl*e could have evolved when sawfish evolved from shovelnose ray-like ancestors [16]–[18], [27]. The reactions by *G. typus* to dipoles suspended in the water column leave open the question about whether these animals could attack prey in the water column. However, this might depend on the type of prey and its mobility.

The display of *spiral* towards the inactive dipole presented in the water column by *G. typus* is clearly distinguished from the display of *spiral* towards the active dipole. When spiralling around the active dipole, the head of the ray remains in contact with the end of the dipole, where the centre of the electric field is located. When spiralling around the inactive dipole, the shovelnose rays only ever kept in contact with the middle of the plastic tube. This difference may be random, but it may also indicate that shovelnose rays will react to an obstruction in the water and investigate it, however, if the obstruction is coupled with an electric field, this will be the centre of investigation. Nevertheless, both *repeated bumps* and *spiral* were quite erratic displays and so no further conclusions can be drawn.

There are two possible explanations for shovelnose rays not clearly reacting to electric dipoles presented in the water column: Firstly, shovelnose rays may have lacked the necessary motivation. As the field strengths were comparable to those that were readily attacked on the substrate, this would indicate that the distance to the substrate was unnaturally large for shovelnose rays. Secondly, the stimulus in the water column may be not as easily detectable as a stimulus on the substrate, as shovelnose rays possess fewer ampullary pores dorsally than ventrally [20]. Both explanations are strengthened by the fact that giant shovelnose rays feed mainly on brachyuran crabs and penaeid shrimp [23].

The ampullary pore counts of *P. microdon* are twice as high as in any other species of sawfish, and are also some of the highest in elasmobranchs [21]. *Pristis microdon* are thus considered electroreception specialists [21]. As the visibility in the natural habitat of juvenile freshwater sawfish in Australia can fall below 10 cm [42], and sawfish can retract their eyes both during feeding and in response to electric dipoles [27], we assume that electroreception also guides the final strike during prey capture in wild freshwater sawfish.

### Behavioural Detection Threshold of Electric Field

The median electric field strength PIR of freshwater sawfish lies well within values reported for other elasmobranchs (*Spyrna lewini* 25.2 nVcm^−1^, *Carcharhinus plumbeus* 30.0 nVcm^−1^ [10], *Sphyrna tiburo* 47.0 nVcm^−1^ [11], *Dasyatis sabina* 5 nVcm^−1^ [43], reviewed in [12]). However, the present study finds that the field strength at PIR does not differ between different size classes of *P. microdon,* although it is assumed that the ampullary sensitivity increases with the canal length [30]. A possible explanation is that the field strength at PIR only indicates the detection threshold and not the actual sensitivity of ampullary canals.

The ‘enhanced electroreception hypothesis’ by Kajiura and Holland [10] predicts that the lateral expansion of the hammerhead cephalofoil provides sphyrnid sharks with both an expanded search area and increased sensitivity towards electric fields compared to similar sized carcharhinid sharks. This hypothesis can be modified to fit the present study in that the elongated rostrum of sawfish has expanded the search area into the water column and increased the sensitivity of the electroreceptive system with an increase in canal length, compared to the closely-related shovelnose rays. Moreover the high density of electroreceptors that are evenly spread out along the whole length of the rostrum (both ventrally and dorsally) [21] provides freshwater sawfish with high electroreceptive resolution.

In keeping with the findings of McGowan and Kajiura [43], there were no significant differences in electric field strength at PIR in saltwater and brackish water treatments for juvenile freshwater sawfish. The electroreceptive sensitivity of *Dasyatis sabina* is reduced in freshwater compared to saltwater and brackish water, although the strength of the electric field in freshwater is increased [43]. This may be due to the increased electric background noise levels in fresh water [14]. As freshwater sawfish use freshwater and brackish waters as nurseries [18], [44], [45], while adults prefer marine habitats, it would be interesting to repeat our experiments in freshwater. This was not possible in the present study due to husbandry constraints.

The strength of the electric field at PIR differed significantly between the two species of shovelnose rays. *A. rostrata* reacted at field strengths that are four times higher than the ones that *G. typus* reacted to. There is no morphological explanation for the different sensitivities of the two species, as their ampullae of Lorenzini are quite comparable in morphology, distribution and number [20]. Therefore, a difference in motivation is suggested, which may be related to the natural electric fields of the species’ prey items, but this remains to be tested. The median electric field strength at PIR for reactions by *G. typus* towards Ag/AgCl electrodes was even more than twenty times higher than for reactions towards salt bridge electrodes. The direct contact between the coated silver pins and seawater of Ag/AgCl electrodes may have caused polarisation currents that shovelnose rays could have detected. This finding underlines the importance of using the same methods as other authors when making comparisons between species.

This paper adds to our knowledge on the behavioural thresholds of elasmobranchs in response to weak electric fields by expanding the species and families tested. If applied in the captive environment, the results of our study could provide an environmental enrichment to rays that are normally fed with dead prey, which lacks any electrical stimulus. The electroreceptive stimulus presented in the water column should be of particular interest to aquaria keeping sawfish, as the behavioural responses of juvenile freshwater sawfish towards these prey-simulating stimuli differ significantly from the behavioural responses elicited by dipoles located on the bottom of the tank. Moreover, our findings show that sawfish may not be benthic but demersal predators, based on their response towards weak electric fields. As all species of sawfish are Critically Endangered [46] understanding of these species’ predatory niches are essential for population recovery attempts.

## References

[1] Kalmijn AJ (1974) The detection of electric fields from inanimate and animate sources other than electric organs. In: Fessard A, editor. Electroreceptors and other specialized receptors in lower vertebrates. Berlin Heidelberg: Springer. 147–200.

[2] Klimley PA (1993) Highly directional swimming by scalloped hammerhead sharks, *Sphyrna lewini*, and subsurface irradiance, temperature, bathymetry and geomagnetic field. Mar Biol 117: 1–22.

[3] Johnson CS, Scronce BL, McManus MW (1984) Detection of DC electric dipoles in background fields by the nurse shark. J Comp Physiol A Sens Neur Behav Physiol 155: 681–687.

[4] Tricas TC, Michael SW, Sisneros JA (1995) Electrosensory optimization to conspecific phasing signals for mating. Neurosci Lett 202: 129–132. 8787848 10.1016/0304-3940(95)12230-3

[5] Sisneros JA, Tricas TC, Luer CA (1998) Response properties and biological function of the skate electrosensory system during ontogeny. J Comp Physiol A Sens Neur Behav Physiol 183: 87–99. 10.1007/s003590050237 9691481

[6] Nelson M (2005) Target Detection, Image Analysis, and Modeling. In: Bullock TH, Hopkins C, Popper AN, Fay RR, editors. Electroreception. New York: Springer. 290–317.

[7] Bodznick DA, Montgomery JC, Tricas TC (2003) Electroreception: Extracting behaviourally important signals from noise. In: Collin SP, Marshall NJ, editors. Sensory processing in aquatic environments. New York: Springer Verlag. 389–403.

[8] Blonder BI, Alvezion WS (1988) Prey discrimination and electroreception in the stingray *Dasyatis sabina* . Copeia 1: 33–36.

[9] Haine OS, Ridd PV, Rowe RJ (2001) Range of electrosensory detection of prey by *Carcharhinus melanopterus* and *Himantura granulata* . Mar Freshw Res 52: 291–296.

[10] Kajiura SM, Holland KN (2002) Electroreception in juvenile scalloped hammerhead and sandbar sharks. J Exp Biol 205: 3609–3621. 12409487 10.1242/jeb.205.23.3609

[11] Kajiura SM (2003) Electroreception in neonatal bonnethead sharks, *Sphyrna tiburo* . Mar Biol 143: 603–611.

[12] Peters RC, Eeuwes LB, Bretschneider F (2007) On the electrodetection threshold of aquatic vertebrates with ampullary or mucous gland electroreceptor organs. Biol Rev Camb Philos Soc 82: 361–373. 17624959 10.1111/j.1469-185X.2007.00015.x

[13] Hopkins C (2005) Passive Electrolocation and the Sensory Guidance of Oriented Behavior. In: Bullock TH, Hopkins C, Popper AN, Fay RR, editors. Electroreception. New York: Springer. 264–289.

[14] Bodznick DA, Montgomery JC (2005) The Physiology of Low-Frequency Electrosensory Systems. In: Bullock TH, Hopkins C, Popper AN, Fay RR, editors. Electroreception. New York: Springer. 132–153.

[15] Kalmijn AJ (1978) Electric and magnetic sensory world of sharks, skates and rays. In: Hodgson ES, Mathewson RF, editors. Sensory biology of sharks, skates and rays. Arlington, Va. : Department of the Navy, Office of Naval Research. 507–528.

[16] Schaeffer B (1963) Cretaceaous fishes from Bolovia, with comments on pristid evolution. Am Mus Novit 2159: 1–20.

[17] Cappetta H (1974) Sclerorhynchidae nov. fam., pristidae et pristiophoridae: un exemple de parallelisme chez les selachiens. Compt Rend Acad Scie Paris Serie D 278: 225–228.

[18] Wueringer BE, Squire LJ, Collin SP (2009) The biology of extinct and extant sawfish (Batoidea: Sclerorhynchidae and Pristidae). Rev Fish Biol Fish 19: 445–464.

[19] Aschliman NC, Nishida M, Miya M, Inoue JG, Rosana KM, et al (2012) Body plan convergence in the evolution of skates and rays (Chondrichthyes: Batoidea). Mol Phylogenet Evol 63: 28–42. 22209858 10.1016/j.ympev.2011.12.012

[20] Wueringer BE, Tibbetts IR (2008) Comparison of the lateral line and ampullary system of two species of shovelnose ray. Rev Fish Biol Fish 18: 47–64.

[21] Wueringer BE, Peverell SC, Seymour JE, Squire LJ, Kajiura SM, et al (2011) Sensory systems in sawfishes: Part 1 the ampullae of Lorenzini. Brain Behav Evol 78: 139–149. 21829004 10.1159/000329515

[22] Peverell SC (2009) Sawfish (Pristidae) of the Gulf of Carpentaria, Queensland, Australia. James Cook University, MSc Thesis.

[23] Vaudo JJ, Heithaus MR (2011) Dietary niche overlap in a nearshore elasmobranch mesopredator community. Mar Ecol Prog Ser 425: 247–260.

[24] Kyne PM, Bennett MB (2002) Diet of the eastern shovelnose ray, *Aptychotrema rostrata* (Shaw & Nodder, 1794), from Moreton Bay, Queensland, Australia. Mar Freshwater Res 53: 679–686.

[25] Kalmijn AJ (1972) Bioelectric fields in sea water and the function of the ampullae of Lorenzini in elasmobranch fishes. Scripps Inst Oceanogr Ref Ser 72–83: 1–21.

[26] Barlow GW (1977) Modal action patterns. In: Seboek TA, editor. How animals communicate. Bloomington: Indiana University Press. 98–134.

[27] Wueringer BE, Squire LJ, Kajiura SM, Hart NS, Collin SP (2012) The function of the sawfish’s saw. Curr Biol 22(5): R150–R151. 22401891 10.1016/j.cub.2012.01.055

[28] Kalmijn AJ (1982) Electric and magnetic field detection in elasmobranch fishes. Science 218: 916–918. 7134985 10.1126/science.7134985

[29] Sokal RE, Rohlf FJ (1995) Biometry. The principles and practice of statistics in biological research. New York: W. H. Freeman and Company. 887 p.

[30] Rivera-Vicente AC, Sewell J, Tricas TC (2011) Electrosensitive spatial vectors in elasmobranch fishes: implications for source localization. PLoS One 6: e16008. 21249147 10.1371/journal.pone.0016008 PMC3020962

[31] Hueter RE, Mann DA, Maruska KP, Sisneros JA, Demski LS (2004) Sensory biology of elasmobranchs. In: Carrier CC, Musick JA, Heithaus MR, editors. Biology of sharks and their relatives. CRC Press. 325–268.

[32] Kasumyan AO (2003) The lateral line in fish: Structure, function and role in behaviour. J Ichtyol Res 43: S175–S203.

[33] Kleerekoper H (1978) Chemoreception and the role of its interaction with flow and light perception in the locomotion and orientation of some elasmobranchs. In: Hodgson ES, Mathewson RF, editors. Sensory biology of sharks, skates and rays. Arlington, Va. : Department of the Navy, Office of Naval Research. 269–330.

[34] Kalmijn AJ (1971) The electric sense of sharks and rays. J Exp Biol 55: 371–383. 5114029 10.1242/jeb.55.2.371

[35] Tricas TC (1982) Bioelectric-mediated predation by swell sharks, *Cephaloscyllium ventriosum* . Copeia 1982: 948–952.

[36] Whitehead DL (2002) Ampullary organs and electroreception in freshwater *C. leucas* . J Physiol Paris 96: 391–395. 14692487 10.1016/S0928-4257(03)00017-2

[37] Raschi WG (1984) Anatomical observations on the ampullae of Lorenzini from selected skates and galeoid sharks of the western north Atlantic. The College of William and Mary, PhD thesis: 78 p.

[38] Raschi WG (1986) A morphological analysis of the ampullae of Lorenzini in selected skates (Pisces, Rajoidei). Journal of Morphology 189: 225–247. 10.1002/jmor.1051890303 29929341

[39] Raschi WG (1978) Notes on the gross functional morphology of the ampullary system in two similar species of skates, *Raja erinacea* and *R. ocellata* . Copeia 1: 48–53.

[40] Raschi WG, Adams WH (1988) Depth-related modifications in the electroreceptive system of the euryhaline skate, Raja radiata (Chondrichtyes: Rajidae). Copeia 1: 116–123.

[41] Niehm VH, Carpenter KE (1998) FAO species identification guide for fisheries purposes. The living marine resources of the Western Central Pacific. Volume 2. Cephalopods, crustaceans, holothurians and sharks. Rome: Food and Agricultural Organization of the United Nations.

[42] Wueringer BE (2011) The sensory biology and feeding behaviour of sawfish. University of Queensland, PhD thesis. 277.

[43] McGowan DW, Kajiura SM (2009) Electroreception in the euryhaline stingray, *Dasyatis sabina* . J Exp Biol 212: 1544–1552. 19411548 10.1242/jeb.025247

[44] Mizue K, Hara M (1991) The rectal gland of freshwater sawfish, *Pristis microdon*, and the bull shark, *Carcharhinus leucas*, collected from the Daly and Sepik River. In: Shimizu M, Taniuchi T, editors. Studies on elasmobranchs collected from seven river systems in Northern Australia and Papua New Guinea. Tokyo: Univ Mus Univ Tokyo, Nature and Culture No. 3: 63–69.

[45] Thorburn DC, Morgan DL, Rowland AJ, Gill HS (2007) Freshwater sawfish *Pristis microdon* Latham, 1794 (Chondrichthyes: Pristidae) in the Kimberley region of Western Australia. Zootaxa 1471: 27–41.

[46] IUCN (2006) 2006 IUCN Red List of Threatened Species. Available: http://www.iucnredlist.org. Accessed 2006 Aug 9.

